# Artificial intelligence-based spatial analysis of tertiary lymphoid structures and clinical significance for endometrial cancer

**DOI:** 10.1007/s00262-024-03929-6

**Published:** 2025-02-01

**Authors:** Haruka Suzuki, Kohei Hamada, Junzo Hamanishi, Akihiko Ueda, Ryusuke Murakami, Mana Taki, Rin Mizuno, Koichi Watanabe, Hanako Sato, Yuko Hosoe, Hiroaki Ito, Koji Yamanoi, Hiroyuki Yoshitomi, Nobuyuki Kakiuchi, Ken Yamaguchi, Noriomi Matsumura, Seishi Ogawa, Hideki Ueno, Masaki Mandai

**Affiliations:** 1 Department of Gynecology and Obstetrics, Kyoto University Graduate School of Medicine, 54 Kawahara-cho, Shogoin, Sakyo-ku, Kyoto, 606-8507 Japan; 2 Department of Obstetrics and Gynecology, Kindai University, Osaka, Japan; 3 Department of Pathology, Kyoto University Graduate School of Medicine, Kyoto, Japan; 4 Department of Immunology, Kyoto University Graduate School of Medicine, Kyoto, Japan; 5 Department of Pathology and Tumor Biology, Kyoto University, Kyoto, Japan; 6 Kyoto University Immunomonitoring Center, Kyoto University, Kyoto, Japan; 7 Institute for the Advanced Study of Human Biology, Kyoto University, Kyoto, Japan

**Keywords:** Endometrial cancer, Tertiary lymphoid structure, Immune checkpoint inhibitors, Artificial intelligence, B cell receptor repertoire

## Abstract

With the incorporation of immune checkpoint inhibitors into the treatment of endometrial cancer (EC), a deeper understanding of the tumor immune microenvironment is critical. Tertiary lymphoid structures (TLSs) are considered favorable prognostic factors for EC, but the significance of their spatial distribution remains unclear. B cell receptor repertoire analysis performed using six TLS samples located at various distances from the tumor showed that TLSs in distal areas had more shared B cell clones with tumor-infiltrating lymphocytes. To comprehensively investigate the distribution of TLSs, we developed an artificial intelligence model to detect TLSs and determine their spatial locations in whole-slide images. Our model effectively quantified TLSs, and TLSs were detected in 69% of the patients with EC. We identified them as proximal or distal to the tumor margin and demonstrated that patients with distal TLSs (dTLSs) had significantly prolonged overall survival and progression-free survival (PFS) across multiple cohorts [hazard ratio (HR), 0.56; 95% confidence interval (CI), 0.36–0.88; *p* = 0.01 for overall survival; HR, 0.58; 95% CI, 0.40–0.84; *p* = 0.004 for PFS]. When analyzed by molecular subtype, patients with dTLSs in the copy-number-high EC subtype had significantly longer PFS (HR, 0.51; 95% CI, 0.29–0.91; *p* = 0.02). Moreover, patients with dTLSs had a higher response rate to immune checkpoint inhibitors (87.5 vs. 41.7%) and a trend toward improved PFS. Our findings indicate that the functions and prognostic implications of TLSs may vary with their locations, and dTLSs may serve as prognostic factors and predictors of treatment efficacy. This may facilitate personalized therapy for patients with EC.

**Supplementary Information:**

The online version contains supplementary material available at 10.1007/s00262-024-03929-6.

## Introduction

Endometrial cancer (EC) is among the most common gynecologic malignancies globally [1]. The long-standing standard treatment for EC has been primary surgery followed by chemotherapy or radiotherapy. With genetic evaluations becoming more widespread for EC [2], immunotherapy—particularly with immune checkpoint inhibitors (ICIs)—has been integrated with standard treatment regimens [3–7]. Consequently, a more comprehensive understanding of the tumor immune microenvironment (TiME) is necessary, and developing biomarkers predictive of ICI response is highly desired.

Tertiary lymphoid structures (TLSs) have been widely studied recently. They are B cell-rich formations present in chronic inflammatory conditions such as autoimmune diseases, chronic infections, chronic graft rejection, and various solid tumors [8, 9]. TLSs serve as pivotal sites for tumor immune engagement, initiating inflammatory responses via tumor-infiltrating lymphocytes (TILs). The presence of TLSs has been demonstrated to be associated with favorable outcomes of malignancies such as breast cancer [10], ovarian cancer [11], and melanoma [12]. In the context of EC, our previous study demonstrated that the presence of TLS is associated with a higher number of TILs and better clinical outcomes [13].

Despite extensive research, considerable variability remains in the evaluation of TLSs in the current literature. The definitions of TLS, maturation stages, evaluation methodologies, locations within the TiME (intratumoral, peritumoral, or stromal regions) [14], quantification methods, and the selection of markers (such as CD19, CD20, CD21, and CD23) are different. In 2023, a standardized protocol for assessing TLSs on pathology slides was proposed [15]. However, performing such high-resolution evaluations reproducibly on whole H&E slides is nearly impossible. Additionally, assessing TLSs often requires immunohistochemistry (IHC), which is not routinely included in clinical practice. While recent clinical trials have preserved digitized pathology slides (whole-slide images, WSIs), they typically provide only one representative hematoxylin and eosin (H&E) WSI per case. Therefore, efficient methods are needed for this labor-intensive evaluation of TLSs on H&E slides.

With the rapid progress of digital pathology, convolutional neural networks (CNNs) have been increasingly employed for various tasks, especially in spatial analyses of TiME. We previously established an artificial intelligence (AI)-based spatial assessment pipeline to objectively quantify intraepithelial and stromal TILs [16]. However, few attempts have been made to quantify the spatial distribution of TLSs using only H&E slides [17, 18].

In this study, we aimed to automatically extract TLSs from EC using AI applied solely to H&E slides. By combining the AI-identified TLSs and tumor regions, we achieved precise assessment of the spatial distribution of TLSs. We also aimed to explore the association between these TLS locations and clinical outcomes, such as patient prognosis and the response to ICIs.

## Materials and methods

### Study cohort and datasets

This study included three cohorts: the Kyoto cohort, the ICI cohort, and the TCGA cohort. The Kyoto cohort consisted of 96 patients with EC who were treated at our hospital from 2006 to 2011; this is the same cohort as in our previous report [13]. Tumor tissues were obtained during primary surgery and before any chemotherapy. One representative H&E slide per patient was selected. The slides were digitally scanned with a NanoZoomer Digital Pathology System (Hamamatsu Photonics, Hamamatsu, Japan) at 20× magnification (resolution of 0.5 micron per pixel).

Twenty patients who had undergone primary surgery for EC and uterine carcinosarcoma and were treated with ICIs at Kyoto University Hospital and Kindai University Hospital for recurrence between 2019 and 2023 were included in the ICI cohort. Regarding the patient background, lenvatinib plus pembrolizumab (LEN/PEM) was approved in Japan in December 2021. For cases prior to this date, all patients received PEM-monotherapy, and they were MSI-H (*n* = 4). Post-2022, most cases (unless contraindicated) were treated with LEN/PEM (PEM: *n* = 2, LEN/PEM: *n* = 14). Tumor tissues were obtained during primary surgery and before any chemotherapy. One representative H&E slide per patient was selected.

The dataset from the TCGA-UCEC archive consisted of 505 cases with 566 WSIs available. Of these, prognostic information was available for 463 cases, which were included in the TCGA cohort. Further details of these cohorts are presented in Table 1.

**Table 1:** Clinical characteristics of the patients

Cohort	Kyoto	TCGA	ICI
Age, median (range)	57 (36–89)	64 (13.8)	62 (43–78)
*Stage, n (%)*			
I	60 (62.5)	295 (63.7)	4 (25)
II	3 (3.1)	43 (9.3)	1 (5)
III	26 (27.1)	102 (22.0)	12 (60)
IV	3 (3.1)	23 (5.0)	3 (15)
*Histology, n (%)*			
Endometrioid	69 (71.9)	354 (76.5)	10 (50)
Serous	26 (27.1)	109 (23.5)	1 (5)
Other	1 (1.0)	0 (0.0)	9 (45)
*Molecular subtype, n (%)*			
POLE	22 (22.9)	42 (9.1)	0 (0.0)
MSI-H	20 (20.8)	130 (28.1)	7 (35)
CNV-H	21 (21.9)	134 (28.9)	2 (10)
CNV-L	26 (27.1)	136 (29.4)	1 (5)
NA	7 (72.9)	21 (4.5)	10 (50)
Total, *n* (%)	96 (100)	463 (100)	20 (100)

*TCGA* The Cancer Genome Atlas, *ICI* immune checkpoint inhibitor, *POLE* polymerase epsilon, *MSI-H* microsatellite instability-high, *CNV-H* copy-number variant-high, *CNV-L* copy-number variant-low, *NA* not available

### Immunohistochemistry

CD21 IHC was performed using a standard protocol. Briefly, paraffin-embedded tumor blocks were sectioned at 4 µm thickness and stained with a mouse monoclonal anti-CD21 antibody (Novus Biologicals. CO, USA). Detailed information is provided in Supplementary Method. Evaluation of IHC staining was performed by a gynecological pathologist and two gynecological oncologists, as previously described [13].

### Tissue patch generation from WSIs and training data generation

For supervised machine learning applied to pathology tissue slides, WSIs were divided into small patches (tiles) with assigned labels for each tile. The tile size was set to 500 × 500 pixels (500 × 500 µm) at 10× magnification based on the input data size of the CNN model and the optimal magnification for labeling the training data. Normalization of staining was performed using a previously reported method (https://github.com/schaugf/HEnorm_python).

From the 24 WSIs in the Kyoto cohort, we generated image patches containing TLS regions, guided by CD21 IHC staining of adjacent tissue sections. We also created tile images containing tumor and stromal regions from both the Kyoto (24 WSIs) and TCGA (82 WSIs) cohorts. For each tile, we manually assigned three classes of labels, including the tumor, stroma, and TLS. The labeling was conducted by two gynecological oncologists using Labelme software (https://www.labelme.io/) and verified by a gynecological pathologist. A total of 966 image patches, each measuring 500 × 500 pixels, were generated at 10× magnification. Among these, 54 patches contained TLSs, 502 were tumor-dominant patches, and 410 were stroma-dominant patches. These patches were evenly distributed during training.

### Model Parameters and evaluation

For the development of the TLS detection model (TLS model), we employed a DeepLabV3_resnet101 model pretrained on ImageNet [19]. The CNN architecture remained the standard DeepLabV3 framework with a ResNet101 backbone, as commonly implemented in PyTorch. Training was performed over 100 epochs with a learning rate of 0.0001. The performance of the model was evaluated using the Dice coefficient. We had established a TIL detection model using a pan-cancer dataset in our previous study, and we utilized the same model in this study (TIL model) [16].

### Quantitative assessment of TILs/TLSs

Following previous studies [10, 20, 21], we assessed TLSs located up to 5000 µm from the tumor margin. The distance from the tumor invasive margin to the center of each TLS was calculated. For the initial preparation of TLS samples used in BCR repertoire analysis and RNA sequencing, we measured distances visually under a microscope. For the distributional evaluations and survival analyses, we calculated the distance based on AI predictions. We categorized TLSs adjacent to the tumor invasive margin (< 500 µm) as proximal TLSs (pTLSs) and those situated between 500 and 5000 µm as distal TLSs (dTLSs). TLSs smaller than 1000 µm^2^ were excluded from the analysis. As detailed in our previous reports [16], we defined intratumoral TILs (iTILs) as TIL-positive tiles present within the tumor epithelium. The iTIL score was calculated by dividing the area of TIL-positive tiles by the total tumor area.

### B cell receptor repertoire analysis

We investigated the clonal diversity of the immunoglobulin G (IgG) repertoire of B cell receptors (BCRs) in both TLSs and TILs from tumor samples of six patients. Utilizing a next-generation sequencing-based immune repertoire analysis with a proprietary adaptor-ligation PCR technique by Repertoire Genesis Inc. (Osaka, Japan), we microdissected two TLSs per patient. These TLSs were assessed for BCR clones that were common with those found in the TILs in the tumor samples. For each sample, the top 30 BCR clones based on read count were included in the analysis.

### RNA sequencing of TLS samples

Total RNA was extracted from formalin-fixed, paraffin-embedded (FFPE) tumor samples using the DNA/RNA FFPE Kit (Qiagen, Valencia, CA, USA). RNA sequencing of TLS samples from five patients was performed on the Illumina NovaSeq 6000 platform. Detailed information is provided in Supplementary Method.

### Gene expression analysis

RNA sequencing data were available for 461 cases in the TCGA cohort and obtained from the TCGA-UCEC archive. We obtained gene sets of TLS-related signatures from previous reports. These included 12 chemokines [22], CXCL13 [23], plasma cells [24], T follicular helper (Tfh) cells [25], T helper 1 (Th1) cells [25], Th1/B cells [26], and TLS imprint signatures [27], and single-sample Gene Set Enrichment Analysis (ssGSEA) was performed. The cytolytic (CYT) score was calculated as the geometric mean of *GZMA* and *PRF1* expression, as previously described [28]. The T cell-inflamed gene expression profile (GEP) score was calculated as a weighted sum of normalized expression values for 18 genes, as previously described [29]. The genes mentioned above are listed in Supplementary Table S1.

### Targeted-capture sequencing of cancer-associated genes

Ninety-six cases of FFPE or fresh frozen surgical specimens from the Kyoto cohort were analyzed using targeted-capture sequencing. The xGen Custom Hybridization Panel (Integrated DNA Technologies, Inc. IA, USA), designed for 140 genes associated with gynecologic cancers (Supplementary Table S2), and the xGen Copy-Number Variation (CNV) Backbone Hybridization Panel were used, as previously described [30]. Molecular subtypes were determined in the following order based on preliminary assessment using the TCGA dataset: (1) polymerase epsilon (POLE) subtype if missense mutation exists at POLE exonuclease domain (residues 268–471); (2) microsatellite instability-high (MSI-H) subtype if two or more frameshift indels were identified in repetitive sequences consisting of more than three consecutive repeats among the remaining tumors; (3) copy-number variant-high (CNV-H) for those with CNAs affecting more than 20% of the genome; and (4) CNV-low (CNV-L) for the others.

### Statistical analysis

Statistical analyses were performed using Python 3.10. Correlations of continuous variables were assessed using Spearman's rank correlation coefficients. Medians of continuous variables were compared using the Mann‒Whitney U test or the Wilcoxon signed-rank test. Cumulative survival probabilities were calculated using the Kaplan–Meier method, with survival data right-censored at 10 years. Univariate and multivariate Cox proportional hazards regression analyses were conducted to calculate *p*-values, hazard ratios (HRs), and 95% confidence intervals (CIs). Detailed information is provided in Supplementary Method.

## Results

### Spatial distribution of TLS and immunological difference

**Fig. 1:**
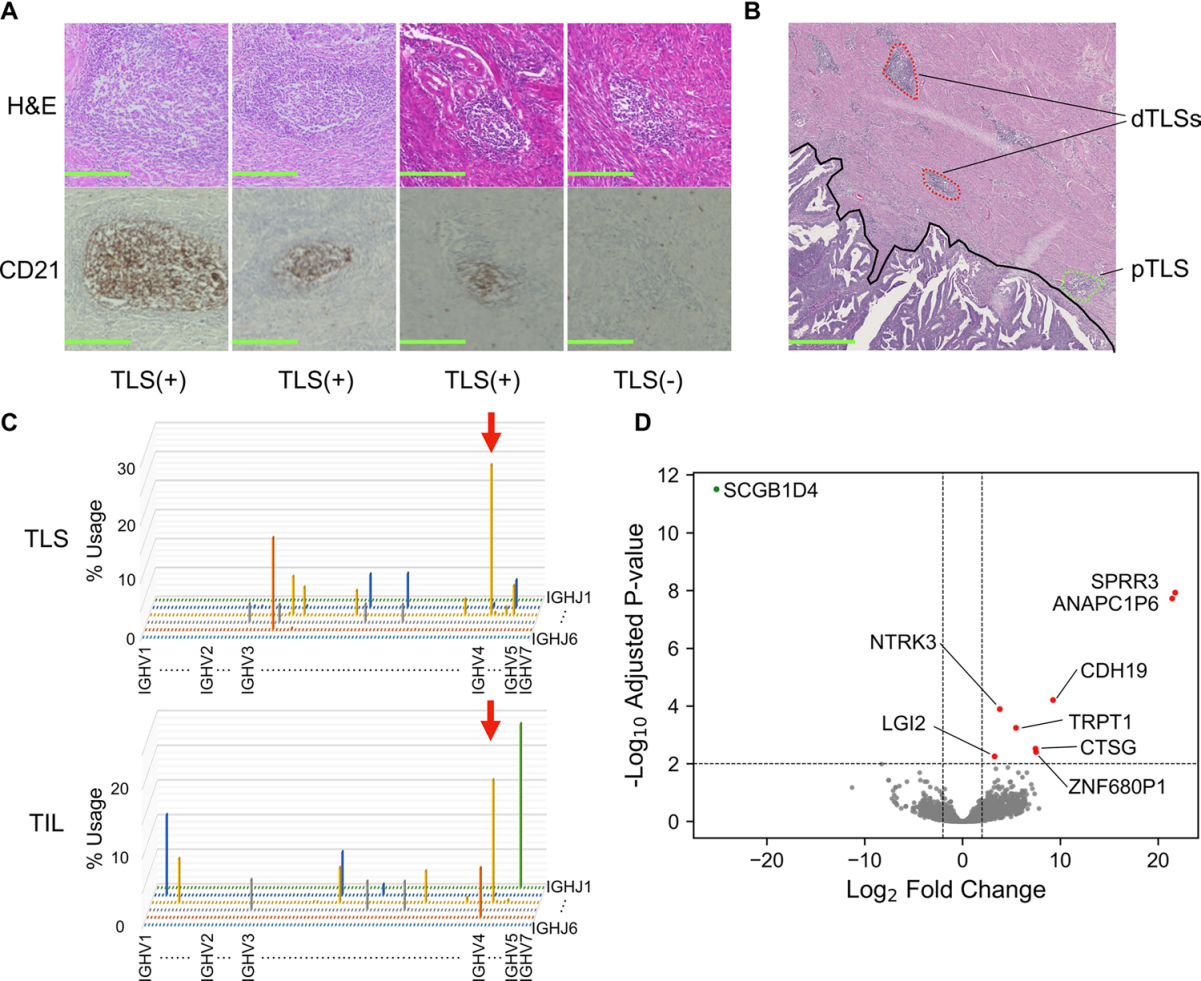
Spatial distribution of TLS and immunological difference. **A** Representative images of H&E slides and CD21 IHC slides. Scale bars at 200 µm. **B** Representative images of H&E slides containing TLSs in the peritumoral area. Tumor invasive margin is indicated by black line. Red and green dotted lines indicate distal and proximal TLSs. Scale bars at 1 mm. **C** B cell receptor repertoire analysis separating TLS and TIL regions. The horizontal axis represents the J gene, the depth represents the V gene, and the vertical axis represents the frequency of usage. Clones indicated by red arrows confirm the same amino acid sequence of the CDR3. IGH VJ Repertoire 3D graph of the representative case with shared clones between the TLS and TIL samples is shown (Case 4). The results of all cases are summarized in Table 2. **D** Differentially expressed genes (DEGs) in the distal tertiary lymphoid structures (dTLS) and proximal TLS (pTLS) samples. Eight genes (in red) were significantly upregulated, and one gene (in green) was significantly downregulated in the dTLSs relative to the pTLSs. H&E, hematoxylin and eosin; IHC, immunohistochemistry; TLS, tertiary lymphoid structure; TIL, tumor-infiltrating lymphocyte; CDR3, complementarity-determining region 3; IGH VJ Repertoire 3D graph, 3-dimension graph of B cell repertoire of VJ lesions in heavy chain of IgG; IGHV, V lesion in heavy chain of IgG; IGHJ, J lesion in heavy chain of IgG

We performed CD21 immunostaining for the Kyoto cohort (Fig. 1A). Sixty cases (69%) were positive for TLSs. Upon detailed examination, we found that some cases had TLSs located only in the immediate vicinity of the tumor margin (< 500 µm), while others had TLSs extending beyond this region (Fig. 1B). We defined the former as proximal TLSs (pTLSs) and the latter as distal TLSs (dTLSs). We selected three cases with multiple pTLSs and three cases with multiple dTLSs, and we microdissected two TLSs from a single slide for each case and conducted BCR repertoire analysis paired with intratumoral TIL regions. Almost no common clones with intratumoral TILs were detected in all three cases with proximal TLSs (pTLSs). However, the amplification of shared clones was observed for all three cases with dTLSs (Fig. 1C, Table 2).

**Table 2:** Results of B cell receptor (BCR) repertoire analysis

TLS ID	Case 1		Case 2		Case 3		Case 4		Case 5		Case 6	
	1	2	1	2	1	2	1	2	1	2	1	2
Distance from tumor edge (µm)	140	240	160	190	300	320	565	850	1230	1880	1000	3760
Total in frame reads	12,954	10,507	6086	20,724	80	354	18,430	11,271	130,044	100,542	12,025	6151
Common clone reads (%)	–	–	–	17 (0.1)	–	1 (0.3)	3651 (19.8)	–	–	40,155 (39.9)	1517 (12.6)	341 (5.5)
Unique common clones	0	0	0	1	0	1	5	0	0	2	4	1

Caption: For each case, two tertiary lymphoid structures (TLSs) were micro-dissected to determine whether they shared clones with the tumor-infiltrating lymphocyte (TIL) region in the tumor. The top 30 clones based on read count in each sample were included in the analysis. The number of common clone reads and unique common clones in the TLS and TIL samples is shown. The location of each TLS was represented as the distance from the tumor invasive margin to the center of the TLS. Cases 4, 5, and 6 were dTLS-positive (gray columns)

To further investigate potential functional differences between TLSs based on their distance from the tumor invasive margin, we performed RNA sequencing on the remaining specimens used in the BCR repertoire analysis. The differentially expressed gene (DEG) analysis identified nine genes, including NTRK3 and CTSG, which are known to be involved in TiME in several cancer types (Fig. 1D). CIBERSORTx was also used to estimate immune cell composition, but no specific trends distinguishing pTLS from dTLS were observed. Individual cases demonstrated more pronounced variation (Supplementary Figure S1A).

### Establishment of AI model to quantify spatial distribution of TLS

**Fig. 2:**
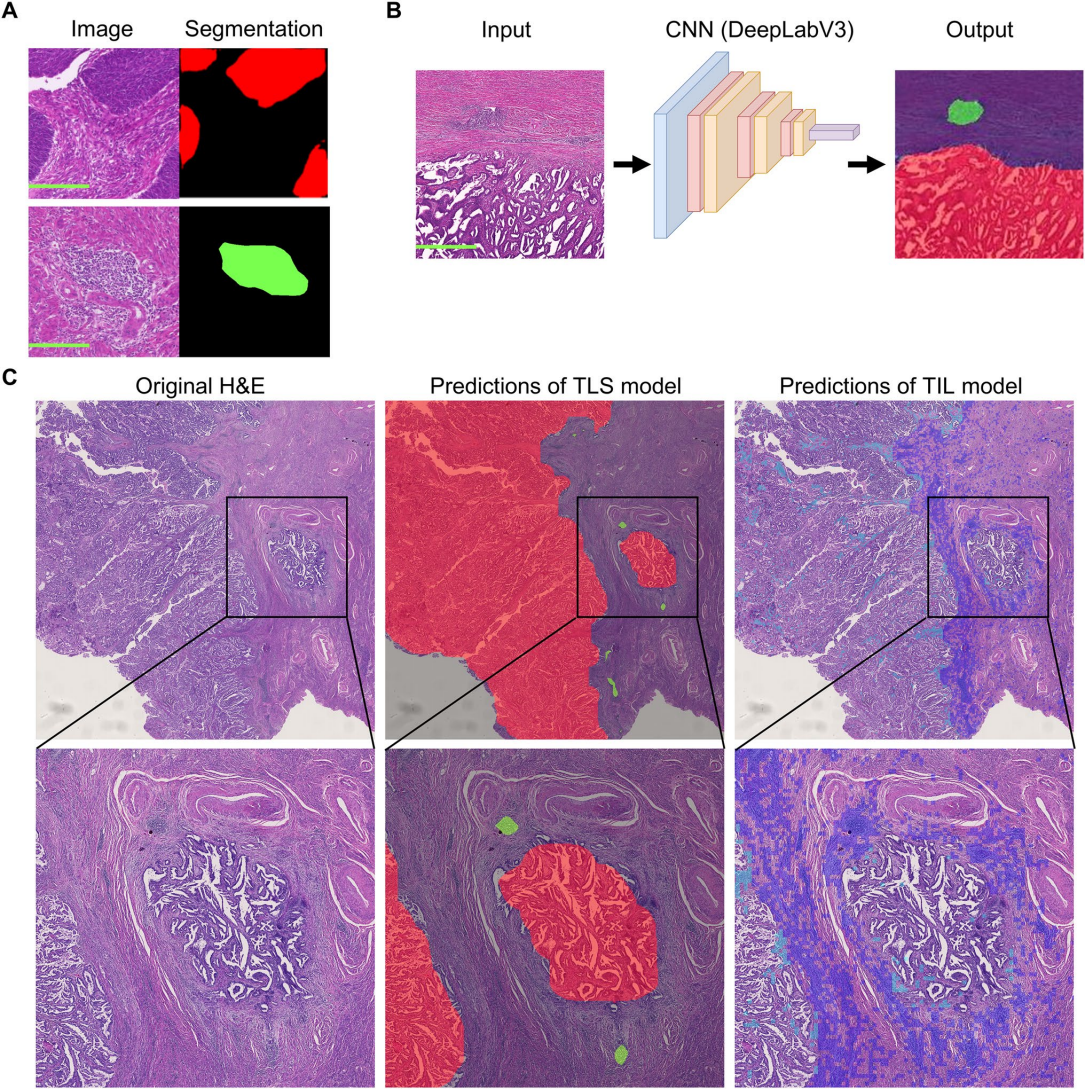
Establishment of AI model to quantify the spatial distribution of TLS. **A** Representative image of patches in the training dataset. Segmented masks are colored red for tumor regions, green for TLS regions, and black for the stroma and background. Scale bars at 200 µm. **B** Concept diagram of the established TLS detection model. Scale bars at 1 mm. **C** Representative images of H&E-stained slides annotated with model predictions. Original H&E-stained slide images, images annotated with predictions from the TLS model (colored green for TLS regions and red for tumor regions), and images annotated with predictions from the TIL model (intratumoral TIL-positive tiles are cyan colored and stromal TIL-positive tiles are blue colored) are shown (upper: low magnification; lower: high magnification). CNN, convolutional neural network

**Fig. 3:**
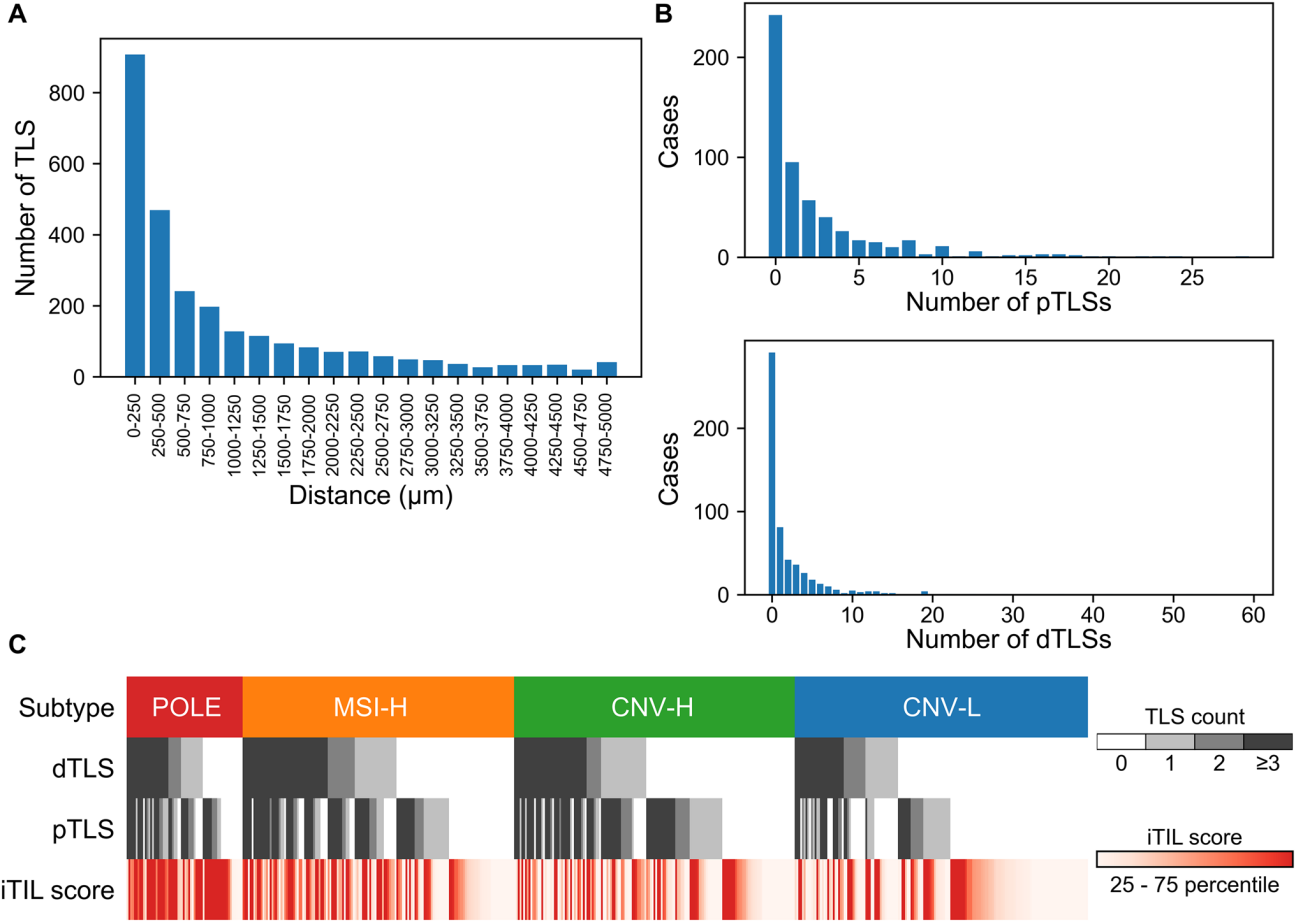
AI-predicted spatial distribution of TLS among molecular subtypes. **A** AI-predicted TLS count stratified by distance from tumor margin. All samples from the TCGA and Kyoto cohort were merged (*n* = 559). **B** AI-predicted dTLS and pTLS counts per case. **C** The association among molecular subtypes, the presence of dTLSs and pTLSs, and the iTIL score. TLS-positive cases are shown in black. Cases with higher iTIL scores are shown in red. POLE, polymerase epsilon-mutated; MSI-H, microsatellite instability-high; CNV-H, copy-number variant-high; CNV-L, copy-number variant-low; iTIL, intratumoral tumor-infiltrating lymphocyte

Based on the above results, we hypothesized that the distance from the tumor margin to the TLSs is important. To investigate TLSs comprehensively, we established an AI model (TLS model) that can distinguish between the tumor and stroma and identify TLSs using our training dataset (Fig. 2A, B). We performed fivefold cross-validation, and the TLS model demonstrated an average Dice coefficient of 0.945 (Supplementary Table S3). By applying the predictions of the model, we delineated the tumor and TLS regions (Fig. 2B, C). Overall, TLSs were positive in 384 cases (69%). We calculated the distance from the tumor margin to each TLS, classifying them into pTLSs and dTLSs. We examined the distribution of TLSs in relation to the distance from the tumor margin. As expected, more TLSs were observed in regions closer to the tumor margin, and some TLSs were still detected in areas up to 5000 µm away from the margin although the number decreased with distance (Fig. 3A). Analysis of the number of TLSs per case revealed that most cases had none or had only a few pTLSs and dTLSs; a large number of TLSs were rarely detected (Fig. 3B).

When analyzed by molecular subtype, the positivity rate of TLSs was relatively lower for the CNV-L (53.1%) than for the POLE, MSI-H, and CNV-H (82.1%, 76.0%, and 74.2%, respectively) subtypes. Regarding dTLSs, the positivity rates were higher for the POLE and MSI-H (65.6% and 56.7%, respectively) than for the CNV-H and CNV-L (47.1% and 35.2%) subtypes (Fig. 3C). However, the number of TLSs did not differ across the molecular subtypes for the TLS-positive cases (Supplementary Figure S2A). We previously reported that TLSs in EC are associated with intratumoral TILs and contribute to favorable clinical outcomes [14]. In another study, we established an AI analysis pipeline that can quantify the density of intratumoral TILs (TIL model) [17]. Therefore, we used this AI-based TIL model to calculate the intratumoral TIL (iTIL) score for each case (Fig. 2C) and found that the cases with TLS had significantly higher iTIL scores (*p* < 0.001) (Fig. 2E, Supplementary Figure S2B). This finding was consistent even when analyzing dTLSs and pTLSs separately, suggesting that TLSs are associated with increased intratumoral TILs (*p* < 0.001 for both dTLSs and pTLSs) (Supplementary Figure S2B).

### Survival analysis based on spatial distribution of TLS and TIL

**Fig. 4:**
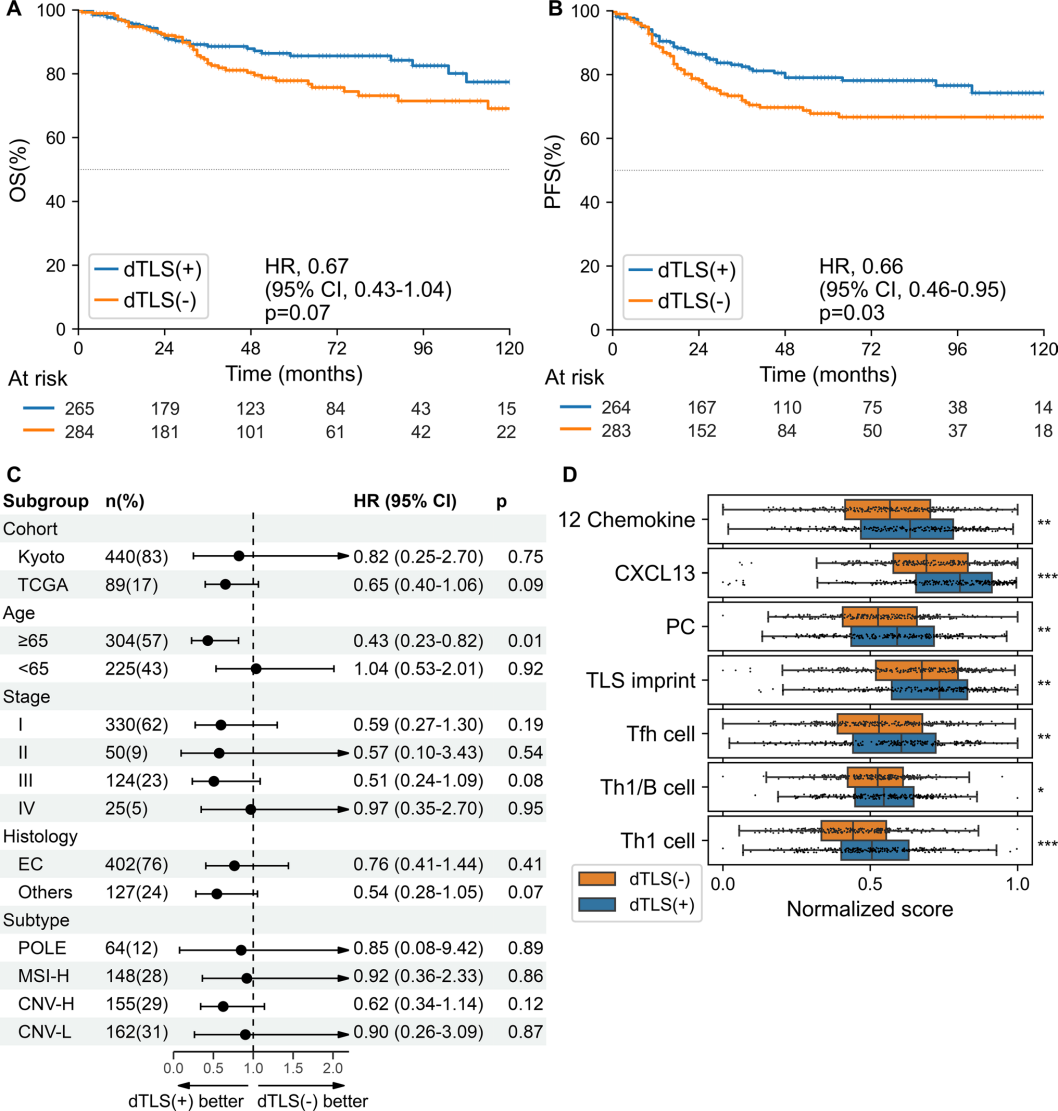
Prognostic impact of dTLSs in patients with endometrial cancer. **A**, **B** Kaplan–Meier curves comparing the (A) overall survival (OS) and (B) progression-free survival (PFS). C**)**Forest plot showing the treatment effect on OS in the subgroup analysis. The vertical line represents the point of no effect. **D** The association between the presence of dTLSs and the ssGSEA scores of TLS-related signatures. The ssGSEA scores were scaled from 0 to 1 using min–max normalization. Boxes in the box plot represent interquartile ranges, and horizontal lines represent the 5–95th percentile ranges, with a notch for the median. P-values were calculated using the Mann‒Whitney U test. HR, hazard ratio; CI, confidence interval; ssGSEA, single-sample Gene Set Enrichment Analysis; *p < 0.05, **p < 0.01, ***p < 0.001

The survival analyses involved 559 patients in the TCGA (*n* = 463) and Kyoto (*n* = 96) cohorts with available prognostic information. We investigated the prognostic impact of TLSs based on their distance from the tumor invasive margin. Previous reports have considered TLSs to be favorable prognostic factors for EC [13, 31]. However, pTLSs, which were located very close to the tumor margin (< 500 µm), showed a trend toward worse prognosis in this study. In contrast, dTLSs, which were located within 500 and 5000 µm from the tumor margin, showed an almost consistent trend toward better prognosis, although these trends were not statistically significant (Supplementary Figure S3A). Therefore, we focused our survival analysis on the dTLSs. Patients with dTLSs had better prognoses than those without them (HR, 0.67; 95% CI, 0.43–1.04; *p* = 0.07 for OS; HR, 0.66; 95% CI, 0.46–0.95; *p* = 0.03 for PFS) (Fig. 4A, B). Multivariable analysis showed that the presence of dTLSs was a significant factor for both OS and PFS (HR, 0.56; 95% CI, 0.36–0.88; *p* = 0.01 for OS; HR, 0.58; 95% CI, 0.40–0.84; *p* = 0.004 for PFS) (Table 3). Subgroup analysis revealed a consistent trend toward better prognosis in the dTLS-positive group across each cohort (Fig. 4C). When analyzed by molecular subtype, patients with dTLSs in the CNV-H group had significantly longer PFS (HR, 0.51; 95% CI, 0.29–0.91; *p* = 0.02) (Supplementary Figures S3B-E). To validate the significance of dTLSs, we calculated the ssGSEA scores for the TLS-related signatures using RNA sequencing data from the TCGA cohort. These signatures are widely used to detect TLSs and serve as a surrogate for clinical significance. Cases predicted to have dTLSs scored significantly higher across all signatures, as expected (Fig. 4D). In contrast, among cases without dTLSs, the ssGSEA scores did not differ significantly between no-TLS- and pTLS-positive groups across all signatures (Supplementary Figure S3F). Additionally, the patients were further divided based on the median iTIL score. Patients with dTLSs and high iTIL scores had favorable prognoses, while those with dTLSs but low iTIL scores had prognoses similar to those of the patients without dTLSs (Supplementary Figure S3G).

**Table 3:** Multivariable Cox proportional hazards regression survival analysis

Variables	Multivariable (OS)			Multivariable (PFS)		
	HR	95% CI	*P* value	HR	95% CI	*P* value
*Stage*						
1	1 (reference)			1 (reference)		
2	1.22	0.47–3.15	0.68	0.99	0.45–2.19	0.98
3	2.54	1.49–4.34	0.006	2.62	1.71–4.01	< 0.001
4	9.63	5.12–18.1	< 0.001	8.37	4.81–14.6	< 0.001
*Age*						
< 65	1 (reference)			1 (reference)		
> = 65	1.74	1.10–2.75	0.02	1.18	0.81–1.72	0.39
*Histology*						
Endometrioid	1 (reference)			1 (reference)		
Others	1.89	1.17–3.06	0.01	1.51	1.01–2.27	0.05
*dTLS*						
Absent	1 (reference)			1 (reference)		
Present	0.56	0.36–0.88	0.01	0.58	0.40–0.84	0.004

*OS* overall survival, *PFS* progression-free survival, *HR* hazard ratio, *CI* confidence interval, *dTLS* distal tertiary lymphoid structure

### ICI response associated with the presence of dTLS

**Fig. 5:**
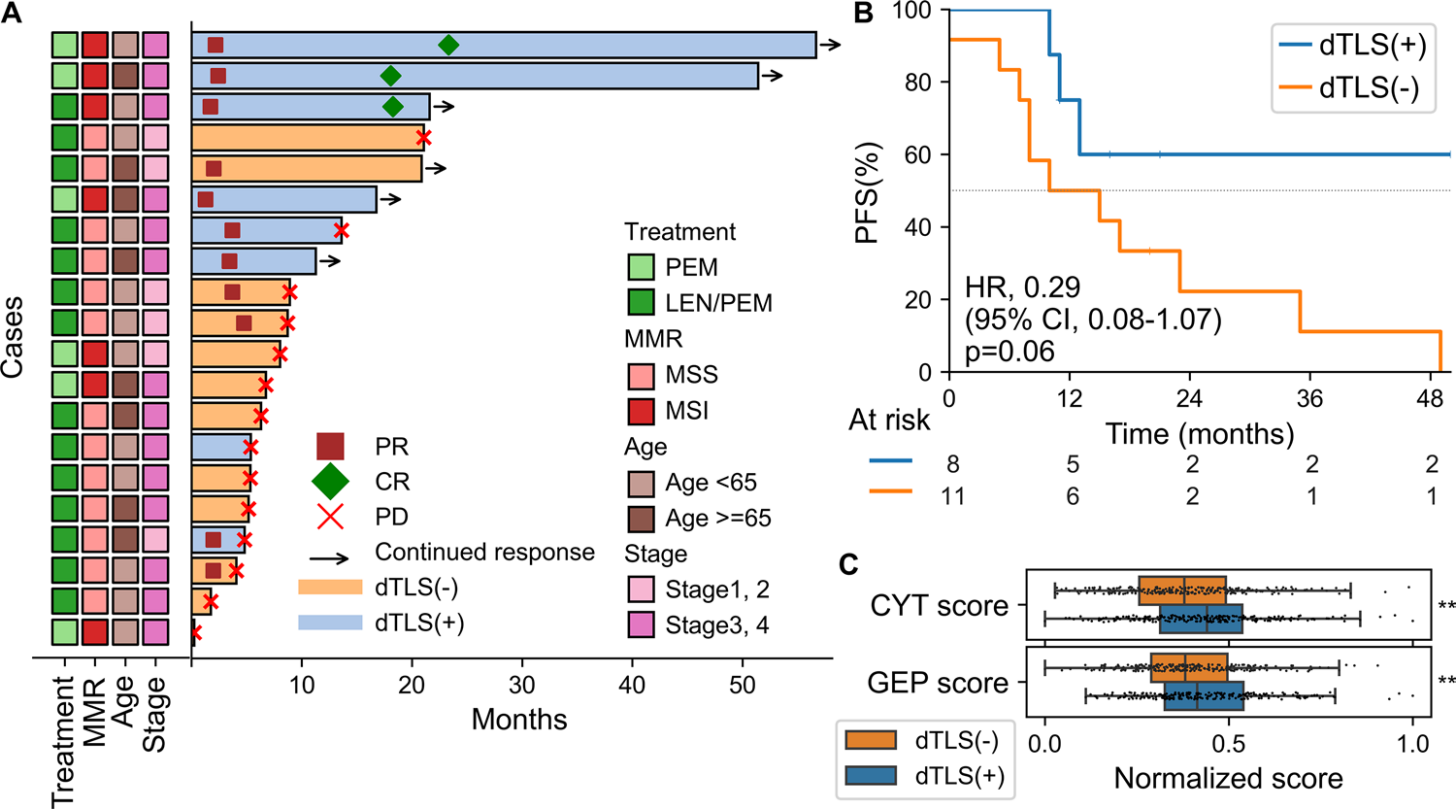
Association between dTLSs and response to immune checkpoint inhibitors (ICIs). **A** Swimmer plot depicting the treatment outcomes of the ICI cohort. The time from the initiation of ICI therapy to disease progression or last follow-up is shown. **B** Kaplan–Meier curves comparing PFS in the ICI cohort. **C** Cytolytic activity (CYT) and T cell-inflamed gene expression profile (GEP) scores of the dTLS-positive and negative groups. Boxes in the box plot represent interquartile ranges, and horizontal lines represent the 5th–95th percentile ranges, with a notch indicating the median. P-values were calculated using the Mann–Whitney U test. **p < 0.01. CR, complete response; PR, partial response; and PD, progressive disease; MMR, mismatch repair; PEM, pembrolizumab; LEN/PEM, pembrolizumab and lenvatinib; MSS, microsatellite stable; MSI, microsatellite instable

Lastly, the association between the presence of dTLS and ICI treatment response was investigated in the ICI cohort with 20 patients (treatment regimens included lenvatinib plus pembrolizumab in 14 cases and pembrolizumab monotherapy in 6 cases). The response rate was higher for the dTLS-positive group (87.5% vs. 41.7%, respectively; Fig. 5A). In MSI-H cases (*n* = 7), four patients achieved a partial response or better and showed a durable response, all of whom were positive for dTLS. In contrast, the other three MSI-H patients, who were negative for dTLS, exhibited progressive disease.

There was a trend toward improved PFS in the dTLS-positive group (HR, 0.29; 95% CI, 0.08–1.07; *p* = 0.06; Fig. 5B). The CYT score, a measure of immune cytolytic activity, and the T cell-inflamed GEP score, an indicator of T cell-activated TiME, have been reported to be associated with ICI response in several cancer types [28, 29]. In the TCGA cohort, gene expression analysis revealed that patients with dTLSs had significantly higher CYT and T cell-inflamed GEP scores (*p* = 0.003 and 0.007, respectively; Fig. 5C).

## Discussion

From the results of our BCR repertoire analysis, we recognized the need to quantitatively evaluate the distance from the tumor invasive margin to TLSs. To address this, we established an AI analysis pipeline that automatically extracts information about TLSs in EC from H&E slides, along with their distance from the tumor margin. Utilizing this approach, we demonstrated consistent prognostic stratification across multiple datasets. We also indicated a potential association with the efficacy of ICIs.

Previous studies have reported that the prognostic impact of TLSs varies depending on their distance from the tumor margin. TLSs can be classified according to their location: tumor nests (T-TLS), peritumoral (P-TLS), and stromal (S-TLS) [14]. The prognostic impact of these TLSs has been inconsistent across different cancers and reports. A favorable prognostic effect of T-TLS has been observed for cholangiocarcinoma, hepatocellular carcinoma (HCC), and metastatic colorectal cancer. However, no significant correlation was found for non-metastatic colorectal cancer [20, 32–34]. P-TLS has been reported to be associated with worse outcomes in cholangiocarcinoma, breast cancer, and metastatic colorectal cancer [10, 20, 32], while with better outcomes for HCC [21]. Notably, in breast cancer, a classification similar to ours—distinguishing between adjacent TLSs (aTLSs) and dTLSs—showed that patients with a higher number of aTLSs had worse prognosis [10]. In EC, S-TLS is relatively rare [13], and P-TLS is predominantly associated with favorable outcomes [13, 31]. Our results indicate that there may be a difference in function and prognostic impact between pTLSs and dTLSs. While we can only speculate, one possibility is that TLSs located too close to the tumor, similar to cancer-metastatic lymph nodes, may be immunologically weakened or exhausted. However, definitive conclusions could not be drawn from our repertoire analysis due to the limited number of cases.

Following the results of the BCR repertoire analysis, we conducted RNA sequencing to compare the pTLSs and dTLSs and identified several notable DEGs. SCGB1D4, which was significantly upregulated in pTLSs, is highly expressed in lymph nodes and has immunological functions, including the regulation of chemotactic migration and invasion [35]. On the other hand, among the genes significantly upregulated in the dTLSs, NTRK3 encodes the tropomyosin receptor kinase C (TRKC) receptor, which, upon binding with neurotrophin-3 (NT-3), can activate MAPK and PI3K/AKT pathways to promote cell growth and differentiation. NTRK3 has been associated with tumor mutation burden and immune infiltration in bladder cancer [36] and with TLSs and TILs in gastrointestinal stromal tumors (GISTs) [37]. Likewise, CTSG encodes Cathepsin G, an immune-related regulatory molecule, which acts as a chemotactic agent for monocytes and stimulates lymphocyte proliferation. Notably, CTSG has been associated with CD4 T cell activation in lung cancer [38]. However, definitive conclusions could not be drawn from our repertoire analysis and RNA sequencing due to the limited number of cases. Since TLS samples were obtained from very small areas, the differences may not be fully detectable by bulk RNA sequencing. Therefore, more detailed analyses involving single-cell RNA sequencing with a larger sample size are warranted.

Given that TLSs are often undetectable in biopsy specimens [9, 17], comprehensive assessment of pathological slides of surgical specimens is required, but this is labor-intensive. To address this issue, several transcriptome-based signatures have been proposed for TLS detection. However, a quantitative relationship between these methods and pathological evaluation has been questioned [22–27]. Furthermore, it is important to develop evaluation techniques that rely solely on H&E staining without IHC to enable larger-scale studies since publicly available datasets typically include only one diagnostic H&E slide. In this study, we elucidated the distribution of TLSs in EC using large-scale data from TCGA after establishing a method to detect TLSs from H&E slides. The higher positivity rates of TLSs in the POLE and MSI-H subgroups were consistent with previous reports [31]. Conversely, the lower TLS positivity rate in the CNV-L subtype aligns with previous reports indicating low TLS-related gene expression in this subtype [39], suggesting that CNV-L represents a more immunologically “cold” environment. Additionally, demonstrating the association with TLS-related signatures further validates the robustness of our method. Given the various attempts to induce TLSs as therapeutic targets [11], the need for quantitative TLS evaluation will persist in the future.

In this study, we demonstrated that dTLSs are consistently associated with favorable prognosis not only in our institutional cohort but also in the TCGA cohort. Moreover, our findings suggest that the prognostic impact of dTLSs may vary among molecular subtypes. For molecular subtyping, a simplified ProMisE classification relying mainly on IHC has been proposed [40]. Horeweg et al. indicated that the prognostic impact of TLSs may vary according to the ProMisE classification of EC [31], highlighting a significant impact in the mismatch repair deficiency (MMRd) group. In contrast, our results did not show a prognostic difference in the MSI-H group, but a significant difference was observed in the CNV-H group. Given the substantial differences in TLS evaluation methods, molecular subtype determination, and patient characteristics, careful interpretation of these results is necessary. The CNV-H group has been reported to be heterogeneous and have immune-hot and immune-cold subclusters, which may partly explain our results [41]. Since the 2023 FIGO staging system for EC incorporates molecular subtypes into the clinical staging [42–44], further investigations guided by the subtypes are needed. Additionally, our sample size was limited, but we presented data on ICI response. Among MSI-H cases that responded to treatment, all were dTLS-positive, suggesting that the presence of dTLS could serve as a more precise predictor of ICI response. While some reports have explored the relationship between TLSs and ICI response for certain cancer types [12, 23, 45], this relationship is not yet well understood. As ICIs are increasingly used as standard treatment for EC[3–7], larger-scale studies are needed in the future.

Our study had several limitations. We demonstrated the consistent prognostic impact of dTLSs, but the functional and biological differences between dTLSs and pTLSs were not thoroughly investigated. Our established AI-based assessment pipeline for TLSs lacked sufficient external validation, as IHC data were not available in public datasets. Additionally, our results indicated an association between TLSs and the response to ICIs, but this finding was inconclusive due to the limited number of cases. Further investigation and well-designed clinical trials with larger number of cases are warranted.

Our AI-based analysis allowed us to reproducibly evaluate the spatial distribution of TLSs on the entire H&E slides. We demonstrated the favorable prognostic value of TLSs by objective quantification and showed the differences according to their spatial distribution. Our findings suggest that TLSs may serve as biomarkers, potentially leading to personalized treatment for patients with EC.

## Supplementary Information

Below is the link to the electronic supplementary material.

Supplementary file1 (DOCX 919 kb)

## Data Availability

No datasets were generated or analyzed during the current study.

## Abbreviations

AI: Artificial intelligence
BCR: B cell receptors
CNA: Copy-number alterations
CNN: Convolutional neural networks
CNV: Copy-number variation
EC: Endometrial cancer
FFPE: Formalin-fixed, paraffin-embedded
GEP: Gene expression profile
H&E: Hematoxylin and eosin
HR: Hazard ratio
ICI: Immune checkpoint inhibitors
IgG: Immunoglobulin G
NA: Not available
OS: Overall survival
PD: Progressive disease
PFS: Progression-free survival
POLE: Polymerase epsilon
TIL: Tumor-infiltrating lymphocyte
TLS: Tertiary lymphoid structure
TPMs: Transcripts per million
VAF: Variant allele frequencies

## References

[1] Siegel RL, Giaquinto AN, Jemal A (2024) Cancer statistics, 2024. CA Cancer J Clin 74(1):12–49. 10.3322/caac.21820 38230766 10.3322/caac.21820

[2] Kandoth C, Schultz N, Cherniack AD, Akbani R, Liu Y, Shen H, Robertson AG, Pashtan I, Shen R, Benz CC, Yau C, Laird PW, Ding L, Zhang W, Mills GB, Kucherlapati R, Mardis ER, Levine DA (2013) Integrated genomic characterization of endometrial carcinoma. Nature 497(7447):67–73. 10.1038/nature12113 23636398 10.1038/nature12113 PMC3704730

[3] Makker V, Colombo N, Casado Herráez A, Santin AD, Colomba E, Miller DS, Fujiwara K, Pignata S, Baron-Hay S, Ray-Coquard I, Shapira-Frommer R, Ushijima K, Sakata J, Yonemori K, Kim YM, Guerra EM, Sanli UA, McCormack MM, Smith AD, Keefe S, Bird S, Dutta L, Orlowski RJ, Lorusso D (2022) Lenvatinib plus pembrolizumab for advanced endometrial cancer. N Engl J Med 386(5):437–448. 10.1056/NEJMoa2108330 35045221 10.1056/NEJMoa2108330 PMC11651366

[4] Bogani G, Monk BJ, Powell MA, Westin SN, Slomovitz B, Moore KN, Eskander RN, Raspagliesi F, Barretina-Ginesta MP, Colombo N, Mirza MR (2024) Adding immunotherapy to first-line treatment of advanced and metastatic endometrial cancer. Ann Oncol 35(5):414–428. 10.1016/j.annonc.2024.02.006 38431043 10.1016/j.annonc.2024.02.006

[5] Mirza MR, Chase DM, Slomovitz BM, dePont CR, Novák Z, Black D, Gilbert L, Sharma S, Valabrega G, Landrum LM, Hanker LC, Stuckey A, Boere I, Gold MA, Auranen A, Pothuri B, Cibula D, McCourt C, Raspagliesi F, Shahin MS, Gill SE, Monk BJ, Buscema J, Herzog TJ, Copeland LJ, Tian M, He Z, Stevens S, Zografos E, Coleman RL, Powell MA (2023) Dostarlimab for primary advanced or recurrent endometrial cancer. N Engl J Med 388(23):2145–2158. 10.1056/NEJMoa2216334 36972026 10.1056/NEJMoa2216334

[6] Eskander RN, Sill MW, Beffa L, Moore RG, Hope JM, Musa FB, Mannel R, Shahin MS, Cantuaria GH, Girda E, Mathews C, Kavecansky J, Leath CA 3rd, Gien LT, Hinchcliff EM, Lele SB, Landrum LM, Backes F, O’Cearbhaill RE, Al Baghdadi T, Hill EK, Thaker PH, John VS, Welch S, Fader AN, Powell MA, Aghajanian C (2023) Pembrolizumab plus chemotherapy in advanced endometrial cancer. N Engl J Med 388(23):2159–2170. 10.1056/NEJMoa2302312 36972022 10.1056/NEJMoa2302312 PMC10351614

[7] Westin SN, Moore K, Chon HS, Lee JY, Thomes Pepin J, Sundborg M, Shai A, de la Garza J, Nishio S, Gold MA, Wang K, McIntyre K, Tillmanns TD, Blank SV, Liu JH, McCollum M, Contreras Mejia F, Nishikawa T, Pennington K, Novak Z, De Melo AC, Sehouli J, Klasa-Mazurkiewicz D, Papadimitriou C, Gil-Martin M, Brasiuniene B, Donnelly C, Del Rosario PM, Liu X, Van Nieuwenhuysen E (2024) Durvalumab plus carboplatin/paclitaxel followed by maintenance durvalumab with or without olaparib as first-line treatment for advanced endometrial cancer: the phase III DUO-E trial. J Clin Oncol: Off J Am Soc Clin Oncol 42(3):283–299. 10.1200/jco.23.02132 10.1200/JCO.23.02132 PMC10824389 37864337

[8] Teillaud JL, Houel A, Panouillot M, Riffard C, Dieu-Nosjean MC (2024) Tertiary lymphoid structures in anticancer immunity. Nat Rev Cancer 24(9):629–646. 10.1038/s41568-024-00728-0 39117919 10.1038/s41568-024-00728-0

[9] Fridman WH, Meylan M, Pupier G, Calvez A, Hernandez I, Sautès-Fridman C (2023) Tertiary lymphoid structures and B cells: an intratumoral immunity cycle. Immunity 56(10):2254–2269. 10.1016/j.immuni.2023.08.009 37699391 10.1016/j.immuni.2023.08.009

[10] Sofopoulos M, Fortis SP, Vaxevanis CK, Sotiriadou NN, Arnogiannaki N, Ardavanis A, Vlachodimitropoulos D, Perez SA, Baxevanis CN (2019) The prognostic significance of peritumoral tertiary lymphoid structures in breast cancer. Cancer Immunol Immunother: CII 68(11):1733–1745. 10.1007/s00262-019-02407-8 31598757 10.1007/s00262-019-02407-8 PMC11028375

[11] Ukita M, Hamanishi J, Yoshitomi H, Yamanoi K, Takamatsu S, Ueda A, Suzuki H, Hosoe Y, Furutake Y, Taki M, Abiko K, Yamaguchi K, Nakai H, Baba T, Matsumura N, Yoshizawa A, Ueno H, Mandai M (2022) CXCL13-producing CD4+ T cells accumulate in the early phase of tertiary lymphoid structures in ovarian cancer. JCI Insight. 10.1172/jci.insight.157215 35552285 10.1172/jci.insight.157215 PMC9309049

[12] Cabrita R, Lauss M, Sanna A, Donia M, Skaarup Larsen M, Mitra S, Johansson I, Phung B, Harbst K, Vallon-Christersson J, van Schoiack A, Lövgren K, Warren S, Jirström K, Olsson H, Pietras K, Ingvar C, Isaksson K, Schadendorf D, Schmidt H, Bastholt L, Carneiro A, Wargo JA, Svane IM, Jönsson G (2020) Tertiary lymphoid structures improve immunotherapy and survival in melanoma. Nature 577(7791):561–565. 10.1038/s41586-019-1914-8 31942071 10.1038/s41586-019-1914-8

[13] Qin M, Hamanishi J, Ukita M, Yamanoi K, Takamatsu S, Abiko K, Murakami R, Miyamoto T, Suzuki H, Ueda A, Hosoe Y, Horie A, Yamaguchi K, Mandai M (2022) Tertiary lymphoid structures are associated with favorable survival outcomes in patients with endometrial cancer. Cancer Immunol Immunother: CII 71(6):1431–1442. 10.1007/s00262-021-03093-1 34689225 10.1007/s00262-021-03093-1 PMC9123039

[14] Schumacher TN, Thommen DS (2022) Tertiary lymphoid structures in cancer. Science 375 (6576):eabf9419. 10.1126/science.abf9419 10.1126/science.abf9419 34990248

[15] Vanhersecke L, Bougouin A, Crombé A, Brunet M, Sofeu C, Parrens M, Pierron H, Bonhomme B, Lembege N, Rey C, Velasco V, Soubeyran I, Begueret H, Bessede A, Bellera C, Scoazec JY, Italiano A, Fridman CS, Fridman WH, Le Loarer F (2023) Standardized pathology screening of mature tertiary lymphoid structures in cancers. Lab Investig J Tech Methods Pathol 103(5):100063. 10.1016/j.labinv.2023.100063 10.1016/j.labinv.2023.100063 36801637

[16] Hamada K, Murakami R, Ueda A, Kashima Y, Miyagawa C, Taki M, Yamanoi K, Yamaguchi K, Hamanishi J, Minamiguchi S, Matsumura N, Mandai M (2024) A deep learning-based assessment pipeline for intraepithelial and stromal tumor-infiltrating lymphocytes in high-grade serous ovarian carcinoma. Am J Pathol 194(7):1272–1284. 10.1016/j.ajpath.2024.02.016 38537936 10.1016/j.ajpath.2024.02.016

[17] Barmpoutis P, Di Capite M, Kayhanian H, Waddingham W, Alexander DC, Jansen M, Kwong FNK (2021) Tertiary lymphoid structures (TLS) identification and density assessment on H&E-stained digital slides of lung cancer. PLoS ONE 16(9):e0256907. 10.1371/journal.pone.0256907 34555057 10.1371/journal.pone.0256907 PMC8460026

[18] Hu J, Coleman K, Zhang D, Lee EB, Kadara H, Wang L, Li M (2023) Deciphering tumor ecosystems at super resolution from spatial transcriptomics with TESLA. Cell Syst 14(5):404-417.e404. 10.1016/j.cels.2023.03.008 37164011 10.1016/j.cels.2023.03.008 PMC10246692

[19] Chen L-C, Zhu Y, Papandreou G, Schroff F, Adam H. Encoder-decoder with atrous separable convolution for semantic image segmentation. In, Cham, 2018. Computer Vision – ECCV 2018. Springer International Publishing, pp 833–851

[20] Ding GY, Ma JQ, Yun JP, Chen X, Ling Y, Zhang S, Shi JY, Chang YQ, Ji Y, Wang XY, Tan WM, Yuan KF, Yan B, Zhang XM, Liang F, Zhou J, Fan J, Zeng Y, Cai MY, Gao Q (2022) Distribution and density of tertiary lymphoid structures predict clinical outcome in intrahepatic cholangiocarcinoma. J Hepatol 76(3):608–618. 10.1016/j.jhep.2021.10.030 34793865 10.1016/j.jhep.2021.10.030

[21] Li H, Liu H, Fu H, Li J, Xu L, Wang G, Wu H (2021) Peritumoral tertiary lymphoid structures correlate with protective immunity and improved prognosis in patients with hepatocellular carcinoma. Front Immunol 12:648812. 10.3389/fimmu.2021.648812 34122408 10.3389/fimmu.2021.648812 PMC8187907

[22] Coppola D, Nebozhyn M, Khalil F, Dai H, Yeatman T, Loboda A, Mulé JJ (2011) Unique ectopic lymph node-like structures present in human primary colorectal carcinoma are identified by immune gene array profiling. Am J Pathol 179(1):37–45. 10.1016/j.ajpath.2011.03.007 21703392 10.1016/j.ajpath.2011.03.007 PMC3123872

[23] Petitprez F, de Reyniès A, Keung EZ, Chen TW, Sun CM, Calderaro J, Jeng YM, Hsiao LP, Lacroix L, Bougoüin A, Moreira M, Lacroix G, Natario I, Adam J, Lucchesi C, Laizet YH, Toulmonde M, Burgess MA, Bolejack V, Reinke D, Wani KM, Wang WL, Lazar AJ, Roland CL, Wargo JA, Italiano A, Sautès-Fridman C, Tawbi HA, Fridman WH (2020) B cells are associated with survival and immunotherapy response in sarcoma. Nature 577(7791):556–560. 10.1038/s41586-019-1906-8 31942077 10.1038/s41586-019-1906-8

[24] Kroeger DR, Milne K, Nelson BH (2016) Tumor-infiltrating plasma cells are associated with tertiary lymphoid structures, cytolytic T-cell responses, and superior prognosis in ovarian cancer. Clin Cancer Res 22(12):3005–3015. 10.1158/1078-0432.ccr-15-2762 26763251 10.1158/1078-0432.CCR-15-2762

[25] Gu-Trantien C, Loi S, Garaud S, Equeter C, Libin M, de Wind A, Ravoet M, Le Buanec H, Sibille C, Manfouo-Foutsop G, Veys I, Haibe-Kains B, Singhal SK, Michiels S, Rothé F, Salgado R, Duvillier H, Ignatiadis M, Desmedt C, Bron D, Larsimont D, Piccart M, Sotiriou C, Willard-Gallo K (2013) CD4⁺ follicular helper T cell infiltration predicts breast cancer survival. J Clin Invest 123(7):2873–2892. 10.1172/jci67428 23778140 10.1172/JCI67428 PMC3696556

[26] Hennequin A, Derangère V, Boidot R, Apetoh L, Vincent J, Orry D, Fraisse J, Causeret S, Martin F, Arnould L, Beltjens F, Ghiringhelli F, Ladoire S (2016) Tumor infiltration by Tbet+ effector T cells and CD20+ B cells is associated with survival in gastric cancer patients. Oncoimmunology 5(2):e1054598. 10.1080/2162402x.2015.1054598 27057426 10.1080/2162402X.2015.1054598 PMC4801425

[27] Meylan M, Petitprez F, Becht E, Bougoüin A, Pupier G, Calvez A, Giglioli I, Verkarre V, Lacroix G, Verneau J, Sun CM, Laurent-Puig P, Vano YA, Elaïdi R, Méjean A, Sanchez-Salas R, Barret E, Cathelineau X, Oudard S, Reynaud CA, de Reyniès A, Sautès-Fridman C, Fridman WH (2022) Tertiary lymphoid structures generate and propagate anti-tumor antibody-producing plasma cells in renal cell cancer. Immunity 55(3):527-541.e525. 10.1016/j.immuni.2022.02.001 35231421 10.1016/j.immuni.2022.02.001

[28] Rooney MS, Shukla SA, Wu CJ, Getz G, Hacohen N (2015) Molecular and genetic properties of tumors associated with local immune cytolytic activity. Cell 160(1–2):48–61. 10.1016/j.cell.2014.12.033 25594174 10.1016/j.cell.2014.12.033 PMC4856474

[29] Ayers M, Lunceford J, Nebozhyn M, Murphy E, Loboda A, Kaufman DR, Albright A, Cheng JD, Kang SP, Shankaran V, Piha-Paul SA, Yearley J, Seiwert TY, Ribas A, McClanahan TK (2017) IFN-gamma-related mRNA profile predicts clinical response to PD-1 blockade. J Clin Invest 127(8):2930–2940. 10.1172/JCI91190 28650338 10.1172/JCI91190 PMC5531419

[30] Nishimura T, Kakiuchi N, Yoshida K, Sakurai T, Kataoka TR, Kondoh E, Chigusa Y, Kawai M, Sawada M, Inoue T, Takeuchi Y, Maeda H, Baba S, Shiozawa Y, Saiki R, Nakagawa MM, Nannya Y, Ochi Y, Hirano T, Nakagawa T, Inagaki-Kawata Y, Aoki K, Hirata M, Nanki K, Matano M, Saito M, Suzuki E, Takada M, Kawashima M, Kawaguchi K, Chiba K, Shiraishi Y, Takita J, Miyano S, Mandai M, Sato T, Takeuchi K, Haga H, Toi M, Ogawa S (2023) Evolutionary histories of breast cancer and related clones. Nature 620(7974):607–614. 10.1038/s41586-023-06333-9 37495687 10.1038/s41586-023-06333-9 PMC10432280

[31] Horeweg N, Workel HH, Loiero D, Church DN, Vermij L, Léon-Castillo A, Krog RT, de Boer SM, Nout RA, Powell ME, Mileshkin LR, MacKay H, Leary A, Singh N, Jürgenliemk-Schulz IM, Smit V, Creutzberg CL, Koelzer VH, Nijman HW, Bosse T, de Bruyn M (2022) Tertiary lymphoid structures critical for prognosis in endometrial cancer patients. Nat Commun 13(1):1373. 10.1038/s41467-022-29040-x 35296668 10.1038/s41467-022-29040-x PMC8927106

[32] Zhang C, Wang XY, Zuo JL, Wang XF, Feng XW, Zhang B, Li YT, Yi CH, Zhang P, Ma XC, Chen ZM, Ma Y, Han JH, Tao BR, Zhang R, Wang TQ, Tong L, Gu W, Wang SY, Zheng XF, Yuan WK, Kan ZJ, Fan J, Hu XY, Li J, Zhang C, Chen JH (2023) Localization and density of tertiary lymphoid structures associate with molecular subtype and clinical outcome in colorectal cancer liver metastases. J Immunother Cancer. 10.1136/jitc-2022-006425 36759015 10.1136/jitc-2022-006425 PMC9923349

[33] Calderaro J, Petitprez F, Becht E, Laurent A, Hirsch TZ, Rousseau B, Luciani A, Amaddeo G, Derman J, Charpy C, Zucman-Rossi J, Fridman WH, Sautès-Fridman C (2019) Intra-tumoral tertiary lymphoid structures are associated with a low risk of early recurrence of hepatocellular carcinoma. J Hepatol 70(1):58–65. 10.1016/j.jhep.2018.09.003 30213589 10.1016/j.jhep.2018.09.003

[34] Wang Q, Shen X, An R, Bai J, Dong J, Cai H, Zhu H, Zhong W, Chen W, Liu A, Du J (2022) Peritumoral tertiary lymphoid structure and tumor stroma percentage predict the prognosis of patients with non-metastatic colorectal cancer. Front Immunol 13:962056. 10.3389/fimmu.2022.962056 36189233 10.3389/fimmu.2022.962056 PMC9524924

[35] Jackson BC, Thompson DC, Wright MW, McAndrews M, Bernard A, Nebert DW, Vasiliou V (2011) Update of the human secretoglobin (SCGB) gene superfamily and an example of “evolutionary bloom” of androgen-binding protein genes within the mouse Scgb gene superfamily. Hum Genomics 5(6):691–702. 10.1186/1479-7364-5-6-691 22155607 10.1186/1479-7364-5-6-691 PMC3251818

[36] Zhang Z, Yu Y, Zhang P, Ma G, Zhang M, Liang Y, Jiao W, Niu H (2021) Identification of NTRK3 as a potential prognostic biomarker associated with tumor mutation burden and immune infiltration in bladder cancer. BMC Cancer 21(1):458. 10.1186/s12885-021-08229-1 33894748 10.1186/s12885-021-08229-1 PMC8070296

[37] Machado I, Claramunt-Alonso R, Lavernia J, Romero I, Barrios M, Safont MJ, Santonja N, Navarro L, López-Guerrero JA, Llombart-Bosch A (2024) ETV6::NTRK3 fusion-positive wild-type gastrointestinal stromal tumor (GIST) with abundant lymphoid infiltration (TILs and Tertiary Lymphoid Structures): a report on a new case with therapeutic implications and a literature review. Int J Mol Sci 25(7). 10.3390/ijms25073707 10.3390/ijms25073707 PMC11011305 38612518

[38] Yan X, Wei S, Yang Y, Zhao Z, Wu Q, Tang H (2024) CTSG may inhibit disease progression in HIV-related lung cancer patients by affecting immunosuppression. Infect Agents Cancer 19(1):34. 10.1186/s13027-024-00599-y 10.1186/s13027-024-00599-y PMC11290089 39080685

[39] Lin Z, Huang L, Li S, Gu J, Cui X, Zhou Y (2020) Pan-cancer analysis of genomic properties and clinical outcome associated with tumor tertiary lymphoid structure. Sci Rep 10(1):21530. 10.1038/s41598-020-78560-3 33299035 10.1038/s41598-020-78560-3 PMC7725838

[40] Talhouk A, McConechy MK, Leung S, Yang W, Lum A, Senz J, Boyd N, Pike J, Anglesio M, Kwon JS, Karnezis AN, Huntsman DG, Gilks CB, McAlpine JN (2017) Confirmation of ProMisE: a simple, genomics-based clinical classifier for endometrial cancer. Cancer 123(5):802–813. 10.1002/cncr.30496 28061006 10.1002/cncr.30496

[41] Mao M, Jiang F, Han R, Xiang Y (2024) Identification of the prognostic immune subtype in copy-number high endometrial cancer. J Gynecol Oncol 35(1):e8. 10.3802/jgo.2024.35.e8 37857563 10.3802/jgo.2024.35.e8 PMC10792215

[42] Berek JS, Matias-Guiu X, Creutzberg C, Fotopoulou C, Gaffney D, Kehoe S, Lindemann K, Mutch D, Concin N (2023) FIGO staging of endometrial cancer: 2023. Int J Gynaecol Obstet: Off Organ Int Feder Gynaecol Obstet 162(2):383–394. 10.1002/ijgo.14923 10.1002/ijgo.14923 37337978

[43] Berek JS, Matias-Guiu X, Creutzberg C, Fotopoulou C, Gaffney D, Kehoe S, Lindemann K, Mutch D, Concin N (2023) FIGO staging of endometrial cancer: 2023. J Gynecol Oncol 34(5):e85. 10.3802/jgo.2023.34.e85 37593813 10.3802/jgo.2023.34.e85 PMC10482588

[44] Han KH, Park N, Lee M, Lee C, Kim H (2024) The new 2023 FIGO staging system for endometrial cancer: what is different from the previous 2009 FIGO staging system? J Gynecol Oncol 35(5):e59. 10.3802/jgo.2024.35.e59 38302727 10.3802/jgo.2024.35.e59 PMC11390244

[45] Helmink BA, Reddy SM, Gao J, Zhang S, Basar R, Thakur R, Yizhak K, Sade-Feldman M, Blando J, Han G, Gopalakrishnan V, Xi Y, Zhao H, Amaria RN, Tawbi HA, Cogdill AP, Liu W, LeBleu VS, Kugeratski FG, Patel S, Davies MA, Hwu P, Lee JE, Gershenwald JE, Lucci A, Arora R, Woodman S, Keung EZ, Gaudreau PO, Reuben A, Spencer CN, Burton EM, Haydu LE, Lazar AJ, Zapassodi R, Hudgens CW, Ledesma DA, Ong S, Bailey M, Warren S, Rao D, Krijgsman O, Rozeman EA, Peeper D, Blank CU, Schumacher TN, Butterfield LH, Zelazowska MA, McBride KM, Kalluri R, Allison J, Petitprez F, Fridman WH, Sautès-Fridman C, Hacohen N, Rezvani K, Sharma P, Tetzlaff MT, Wang L, Wargo JA (2020) B cells and tertiary lymphoid structures promote immunotherapy response. Nature 577(7791):549–555. 10.1038/s41586-019-1922-8 31942075 10.1038/s41586-019-1922-8 PMC8762581

