# Supplementary material for: Artificial intelligence-based spatial analysis of tertiary lymphoid structures and clinical significance for endometrial cancer
Source: Cancer Immunol Immunother. 2025 Feb 1;74(3):84. doi: 10.1007/s00262-024-03929-6 (PMC11787133; doi:10.1007/s00262-024-03929-6)

**Supplementary Material**

**Supplementary Method**

**Supplementary Table**

**Supplementary Table S1. Gene sets and signatures used in the analyses.**

**Supplementary Table S2. Gynecologic cancer-associated genes for targeted capture sequencing.**

**Supplementary Table S3. The result of five-fold cross-validation of the TLS model.**

**Supplementary Figure**

**Supplementary Figure S1. Gene expression of immune cell composition between dTLS and pTLS**

**Supplementary Figure S2. The relationship between spatial Distribution of TLS and molecular subtype and intratumoral tumor-infiltrating lymphocyte score.**

**Supplementary Figure S3.**  **Prognostic impact of TLS based on special distribution, molecular subtype and combination with intratumoral tumor-infiltrating lymphocyte score.**

**Supplementary Method**

***Immunohistochemistry***

CD21 IHC was performed using a standard protocol. Paraffin-embedded tumor blocks were sectioned at 4 µm thickness and heated at 65 °C for 30 minutes in a tissue-drying oven. Deparaffinization was performed with xylene in three steps of 5 minutes each. The sections were then rehydrated in 99% ethanol for 5 minutes, followed by two 2-minute washes in 99% ethanol and a 2-minute wash in 70% ethanol, then rinsed with water. Antigen retrieval was conducted by treating the sections with diluted proteinase K (New England Biolabs. MA, USA) in Tris-EDTA buffer at room temperature for 15 minutes. All sections were treated with methanol containing 0.3% hydrogen peroxide (H₂O₂) for 10 minutes to block endogenous peroxidase activity. Nonspecific IgG binding was blocked using normal rabbit serum (Nichirei Biosciences Inc., Tokyo, Japan). The sections were then incubated overnight at 4 °C with a mouse monoclonal anti-CD21 antibody (Novus Biologicals. CO, USA). Subsequently, they were stained with corresponding biotinylated secondary antibodies (Nichirei Biosciences Inc.) for 30 minutes, followed by incubation with streptavidin-peroxidase solution for another 30 minutes. Diaminobenzidine (DAB; Sigma-Aldrich #D4418, MO, USA) was used to visualize peroxidase activity, and hematoxylin served as a counterstain. Finally, the sections were dehydrated through graded alcohols and xylene before coverslips were applied.

***RNA sequencing***

Total RNA was extracted from formalin-fixed, paraffin-embedded (FFPE) tumor samples using the DNA/RNA FFPE Kit (Qiagen, Valencia, CA, USA). RNA sequencing of TLS samples from five patients was performed on the Illumina NovaSeq 6000 platform with paired-end 150 bp reads, generating approximately 9 gigabases or 60 million reads (30 million read pairs) per sample. Raw reads were trimmed and preprocessed using fastp (version 0.23.2). The processed reads were aligned to the human reference genome GRCh38 using STAR (version 2.7.10a) and quantified with RSEM (version 1.3.1). Differentially expressed gene (DEG) analysis was conducted using the R package DESeq2 (version 1.36.0). After normalization to transcripts per million (TPM), the relative abundance of 22 immune cell types in each sample was estimated using CIBERSORTx (<http://cibersortx.stanford.edu/>).

***Targeted-capture sequencing of cancer-associated genes***

A total of 96 cases of FFPE or fresh frozen surgical specimens from the Kyoto cohort were analyzed using targeted-capture sequencing. This was performed with the xGen Custom Hybridization Panel (Integrated DNA Technologies, Inc. IA, USA), designed for 140 genes associated with gynecologic cancers (Supplementary Table S2), and the xGen Copy Number Variation (CNV) Backbone Hybridization Panel. Mutation calling was conducted using Genomon2, following established protocols. Variants were excluded as germline mutations or sequencing artifacts if they did not meet the following criteria: (1) sufficient sequencing depth (coverage of eight reads or more); (2) variant allele frequencies (VAFs) greater than 0.02 with at least four variant reads; and (3) EBCall *p*-values less than 10^-4^ and fewer than five variant reads in the normal control panel. Copy-number alterations (CNAs) were evaluated based on the sequencing data using CNACS. Molecular subtypes were determined in the following order based on preliminary assessment using the TCGA dataset: (1) polymerase epsilon (POLE) subtype if missense mutation exists at POLE exonuclease domain (residues 268-471) (2) microsatellite instability-high (MSI-H) subtype if two or more frameshift indels were identified in repetitive sequences consisting of more than three consecutive repeats (3) among the remaining tumors, those with CNAs affecting over 20% of the genome were classified as copy-number variant-high (CNV-H), and the others were labeled CNV-low (CNV-L).

***Statistical Analysis***

For Kaplan-Meier analyses, patients were divided based on dTLS positivity, and univariate analysis was performed. Multivariate analyses included additional clinical factors such as age, clinical stage, and histology. Overall survival (OS) and progression-free survival (PFS) were defined as the time from initial treatment to death and recurrence or last follow-up, respectively. For the ICI cohort, PFS was defined as the time from initiation of ICI treatment to disease progression or last follow-up. Two-sided p-values less than 0.05 were considered statistically significant in all analyses.

**Supplementary Table**

**Supplementary Table S1. Gene sets and signatures used in the analyses.**

| Signatures | | | | | | | | Scores | |
| --- | --- | --- | --- | --- | --- | --- | --- | --- | --- |
| 12 chemokines | CXCL13 | Plasma cells | Tfh cells | Th1 cells | Th1/B cells | TLS imprint | | CYT | GEP |
| *CCL2* | *CXCL13* | *TNFRSF17* | *CD200* | *CCND2* | *CD4* | *IGHA1* | *IL-7R* | *GZMA* | *CCL5* |
| *CCL3* |  | *IGJ* | *CXCL13* | *CD38* | *CCR5* | *IGHG1* | *CXCL12* | *PRF1* | *CD27* |
| *CCL4* |  |  | *FBLN7* | *CTLA4* | *CXCR3* | *IGHG2* | *LUM* |  | *CD274* |
| *CCL5* |  |  | *ICOS* | *RAB27A* | *CSF2* | *IGHG3* | *C1QA* |  | *CD276* |
| *CCL8* |  |  | *SGPP2* | *RAB33A* | *IGSF6* | *IGHG4* | *C7* |  | *CD8A* |
| *CCL18* |  |  | *SH2D1A* | *SH2D2A* | *IL2RA* | *IGHGP* | *CD52* |  | *CMKLR1* |
| *CCL19* |  |  | *TIGIT* | *STAT1* | *CD38* | *IGHM* | *APOE* |  | *CXCL9* |
| *CCL21* |  |  | *PDCD1* | *TNFRSF4* | *CD40* | *IGKC* | *PTLP* |  | *CXCR6* |
| *CXCL9* |  |  |  | *IL12RB2* | *CD5* | *IGLC1* | *PTGDS* |  | *HLA-DQA1* |
| *CXCL10* |  |  |  | *SAMD9L* | *MS4A1* | *IGLC2* | *PIM2* |  | *HLA-DRB1* |
| *CXCL11* |  |  |  | *CXCL9* | *SDC1* | *IGLC3* | *DERL3* |  | *HLA-E* |
| *CXCL13* |  |  |  | *IFNG* | *GFI1* | *JCHAIN* |  |  | *IDO1* |
|  |  |  |  |  | *IL1R1* | *CD79A* |  |  | *LAG3* |
|  |  |  |  |  | *IL1R2* | *FCRL5* |  |  | *NKG7* |
|  |  |  |  |  | *IL10* | *MZB1* |  |  | *PDCD1LG2* |
|  |  |  |  |  | *CCL20* | *SSR4* |  |  | *PSMB10* |
|  |  |  |  |  | *IRF4* | *XBP1* |  |  | *STAT1* |
|  |  |  |  |  | *TRAF6* | *TRBC2* |  |  | *TIGIT* |
|  |  |  |  |  | *STAT5A* |  |  |  |  |

Tfh, T follicular helper cells; Th1, T helper 1 cells, TLS, Tertiary lymphoid structure; CYT, cytolytic activity; GEP, T cell-inflamed gene expression profile

**Supplementary Table S2. Gynecologic cancer-associated genes for targeted capture sequencing.**

| *ACVR1* | *CCNE1* | *EMSY* | *IFNGR2* | *MSH6* | *PMS1* | *SOS1* |
| --- | --- | --- | --- | --- | --- | --- |
| *AKT1* | *CD274* | *EP300* | *IGF1R* | *MTOR* | *PMS2* | *SOX17* |
| *AKT2* | *CD276* | *EPCAM* | *JAK1* | *MUC4* | *POLD1* | *SOX8* |
| *APC* | *CD58* | *ERBB2* | *JAK2* | *MUTYH* | *POLE* | *SPOP* |
| *ARHGAP35* | *CDK10* | *ERBB3* | *KDR* | *MYC* | *PPP2R1A* | *STAG1* |
| *ARID1A* | *CDK12* | *ESRP1* | *KIT* | *MYCL* | *PTEN* | *STAG2* |
| *ARID1B* | *CDKN1A* | *FAT1* | *KMT2B* | *MYCN* | *RAD51C* | *STAT3* |
| *ARID5B* | *CDKN1B* | *FBXW7* | *KMT2C* | *NF1* | *RAD51D* | *STK11* |
| *ATM* | *CDKN2A* | *FGFR1* | *KMT2D* | *NFE2L2* | *RASA1* | *TAF1* |
| *ATR* | *CDKN2B* | *FGFR2* | *KRAS* | *NRAS* | *RB1* | *TAP1* |
| *B2M* | *CHD4* | *FGFR3* | *LRP1B* | *NTRK1* | *RMC1* | *TAP2* |
| *BCOR* | *CHEK2* | *FGFR4* | *LZTR1* | *PALB2* | *RNF43* | *TERT* |
| *BMF* | *CREBBP* | *FOXA2* | *MAP3K1* | *PAX8* | *RPL22* | *TET2* |
| *BRAF* | *CTCF* | *GNAS* | *MAX* | *PBRM1* | *RRAS2* | *TGFBR1* |
| *BRCA1* | *CTNNB1* | *HLA-A* | *MECOM* | *PDCD1LG2* | *SETD1B* | *TGFBR2* |
| *BRCA2* | *CUL3* | *HLA-B* | *MED12* | *PHACTR4* | *SHKBP1* | *TP53* |
| *BRIP1* | *CUX1* | *HLA-C* | *MLH1* | *PIK3CA* | *SIN3A* | *U2AF1* |
| *CASP8* | *DICER1* | *HNRNPA2B1* | *MLH3* | *PIK3R1* | *SMAD2* | *ZBTB7B* |
| *CCDC73* | *EGFR* | *HRAS* | *MSH2* | *PIK3R2* | *SMAD4* | *ZFHX3* |
| *CCND1* | *EIF1AX* | *IFNGR1* | *MSH3* | *PLXNB2* | *SMARCA4* | *ZNF217* |

**Supplementary Table S3. The result of five-fold cross-validation of the TLS model.**

| Training fold | Dice value |
| --- | --- |
| 1 | 0.928 |
| 2 | 0.945 |
| 3 | 0.954 |
| 4 | 0.951 |
| 5 | 0.947 |
| Average | 0.945 |

**Supplementary Table S4. Multivariable Cox proportional hazards regression survival analysis on the ICI cohort.**

| Variables | Multivariable (PFS) | | |
| --- | --- | --- | --- |
|  | HR | 95% CI | P value |
| Stage |  | | |
| 1 and 2 | 1 (reference) | | |
| 3 and 4 | 2.51 | 1.50 - 100.1 | 0.02 |
| Age |  | | |
| < 65 | 1 (reference) | | |
| >= 65 | 0.21 | 0.04 - 1.21 | 0.08 |
| Treatment |  | | |
| PEN | 1 (reference) | | |
| LEN/PEN | NA | 0 - inf | 1.00 |
| LVSI |  |  |  |
| Absent | 1 (reference) |  |  |
| Present | 0.57 | 0.15 - 2.18 | 0.41 |
| MMR |  |  |  |
| MSS | 1 (reference) |  |  |
| MSI | NA | 0 - inf | 1.00 |
| dTLS |  |  |  |
| Absent | 1 (reference) |  |  |
| Present | 0.11 | 0.01-0.80 | 0.03 |

**Supplementary Figure**

**Supplementary Figure S1. Gene expression of immune cell composition between dTLS and pTLS**

Estimated immune cell composition of TLS samples using CIBERSORTx. Two TLSs per case were microdissected from a single slide.

**
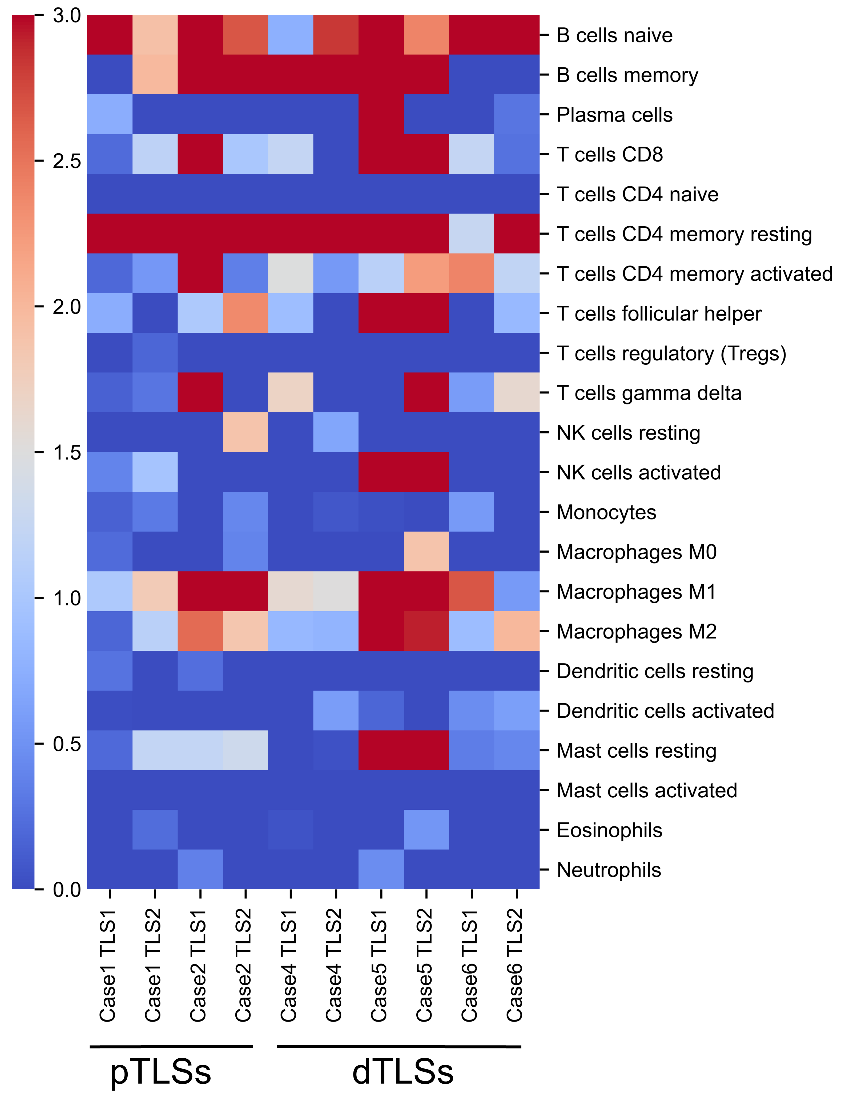
**

**Supplementary Figure S2. The relationship between spatial Distribution of TLS and molecular subtype and intratumoral tumor-infiltrating lymphocyte score**

(A) Number of TLSs by molecular subtype. Cases with no TLSs were excluded from the plot. P-values were calculated using the Mann–Whitney U test and adjusted for multiple comparisons using the Bonferroni method. *p < 0.05. (B) The association between the presence of TLSs and the intratumoral tumor-infiltrating lymphocyte (iTIL) score. P-values were calculated using the Mann–Whitney U test. ***p < 0.001.


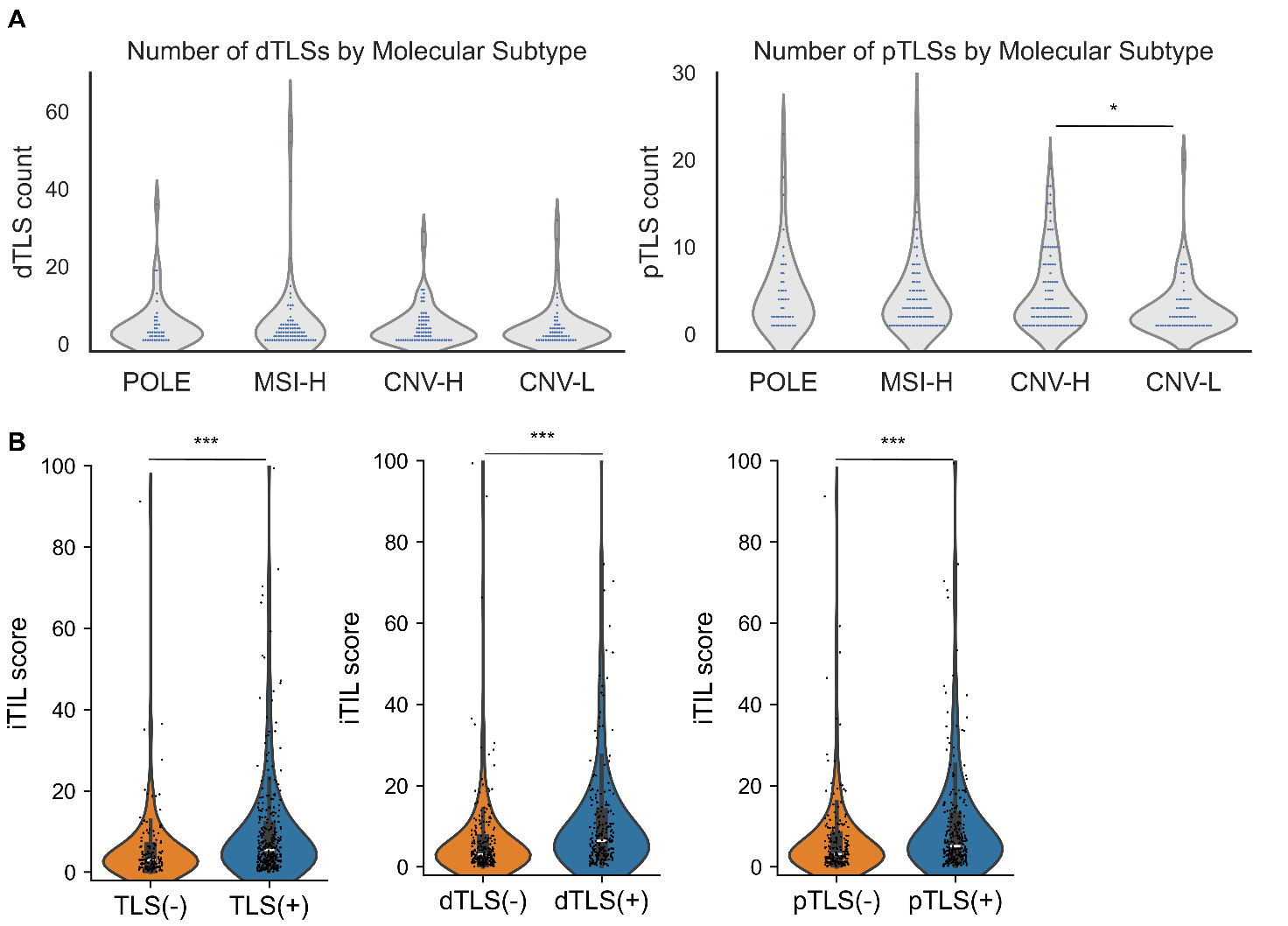


**Supplementary Figure S3. Prognostic impact of TLS based on special distribution, molecular subtype , and combination with tumor-infiltrating lymphocyte score.**

(A) Prognostic impact of TLSs based on their distance from the tumor invasive margin. The vertical line indicates the point of no effect. (B) The association between the presence of TLSs and the iTIL score. (B-E) Prognostic impact of dTLS in each molecular subtype. Kaplan-Meier curves comparing progression-free survival (PFS) in subgroups of (B) polymerase epsilon (POLE)-mutated, (C) microsatellite instability-high (MSI-H), (D) copy-number variant-high (CNV-H), and (E) CNV-low (CNV-L). (F) The association between the presence of pTLSs and the ssGSEA scores of TLS-related signatures in the dTLS negative subgroup. The ssGSEA scores were scaled from 0 to 1 using min-max normalization. Boxes in the box plot represent interquartile ranges, and horizontal lines represent the 5–95th percentile ranges, with a notch for the median. P-values were calculated using the Mann‒Whitney U test. (G) Kaplan-Meier curves of overall survival (OS) according to combination of dTLSs and iTILs. dTLS, distal tertiary lymphoid structure; iTIL, intratumoral tumor-infiltrating lymphocyte, iTIL-high, iTIL score high; iTIL-low, iTIL score low.


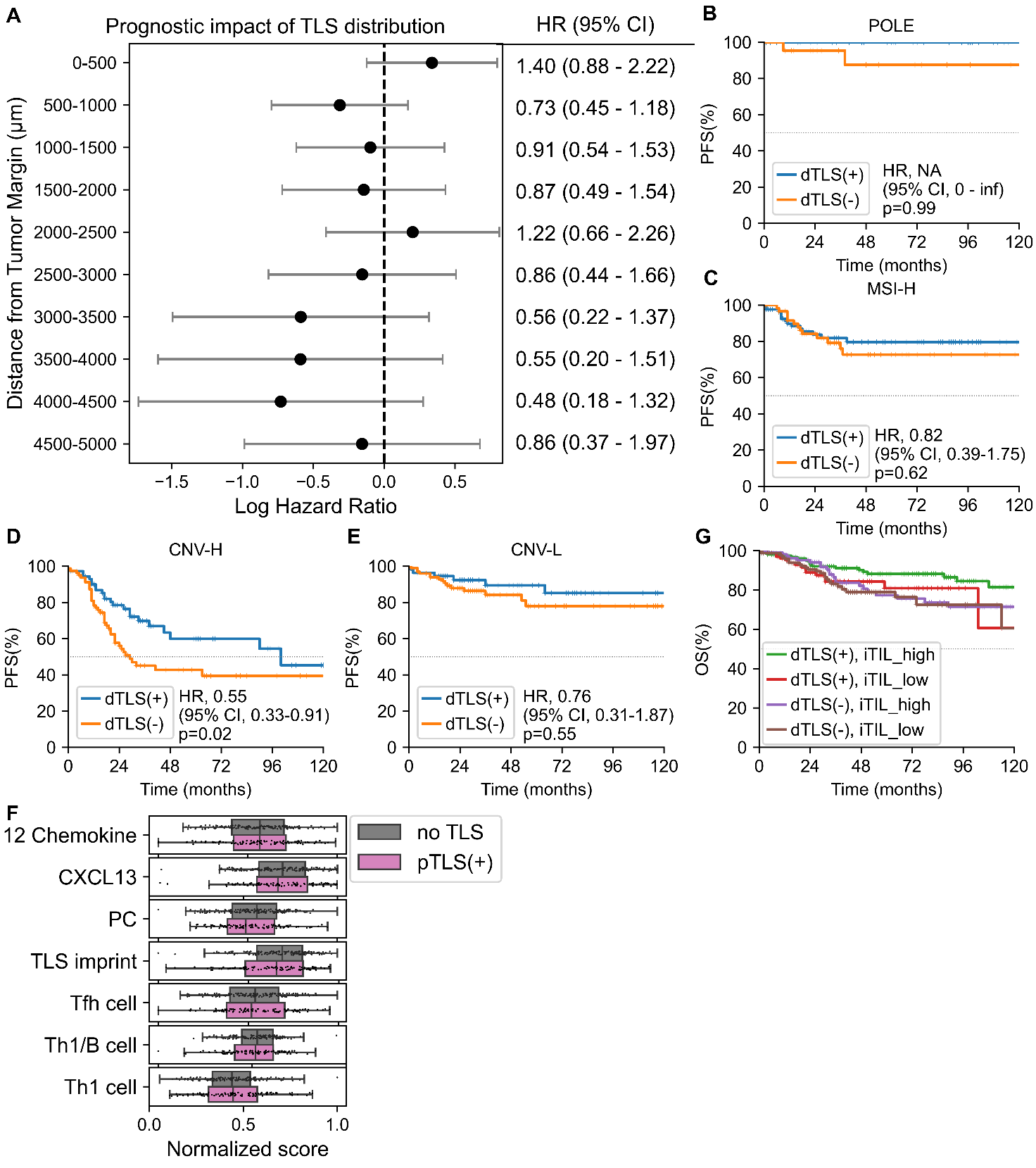

Supplement: Supplementary file 1 — Supplementary file1 (DOCX 919 kb) [file 262_2024_3929_MOESM1_ESM.docx]
